# HHLA2 is a novel prognostic predictor and potential therapeutic target in malignant glioma

**DOI:** 10.3892/or.2022.8473

**Published:** 2022-12-27

**Authors:** Yangzhi Qi, Gang Deng, Pengfei Xu, Huikai Zhang, Fanen Yuan, Rongxin Geng, Hongxiang Jiang, Baohui Liu, Qianxue Chen

Oncol Rep 42: 2309–2322, 2019; DOI: 10.3892/or.2019.7343

Following the publication of this article, an interested reader drew to the authors' attention that, in Fig. 1F on p. 2311 showing a representative high-grade glioma specimen, the data were either duplicated or overlapping with the data featured in Fig. 1D, which showed a low-grade glioma specimen. After having consulted their original data, the authors have realized that the data for Fig. 1D were inadvertently selected incorrectly.

The corrected version of Fig. 1, now showing the correct data for the high-magnification high-grade glioma specimen in Fig. 1F, is shown on the next page. The authors sincerely apologize for the error that was introduced during the preparation of this figure, thank the Editor of *Oncology Reports* for granting them the opportunity to publish a Corrigendum, and are grateful to the reader for alerting them to this issue. The authors also regret any inconvenience that this mistake may have caused.

## Figures and Tables

**Figure 1.**
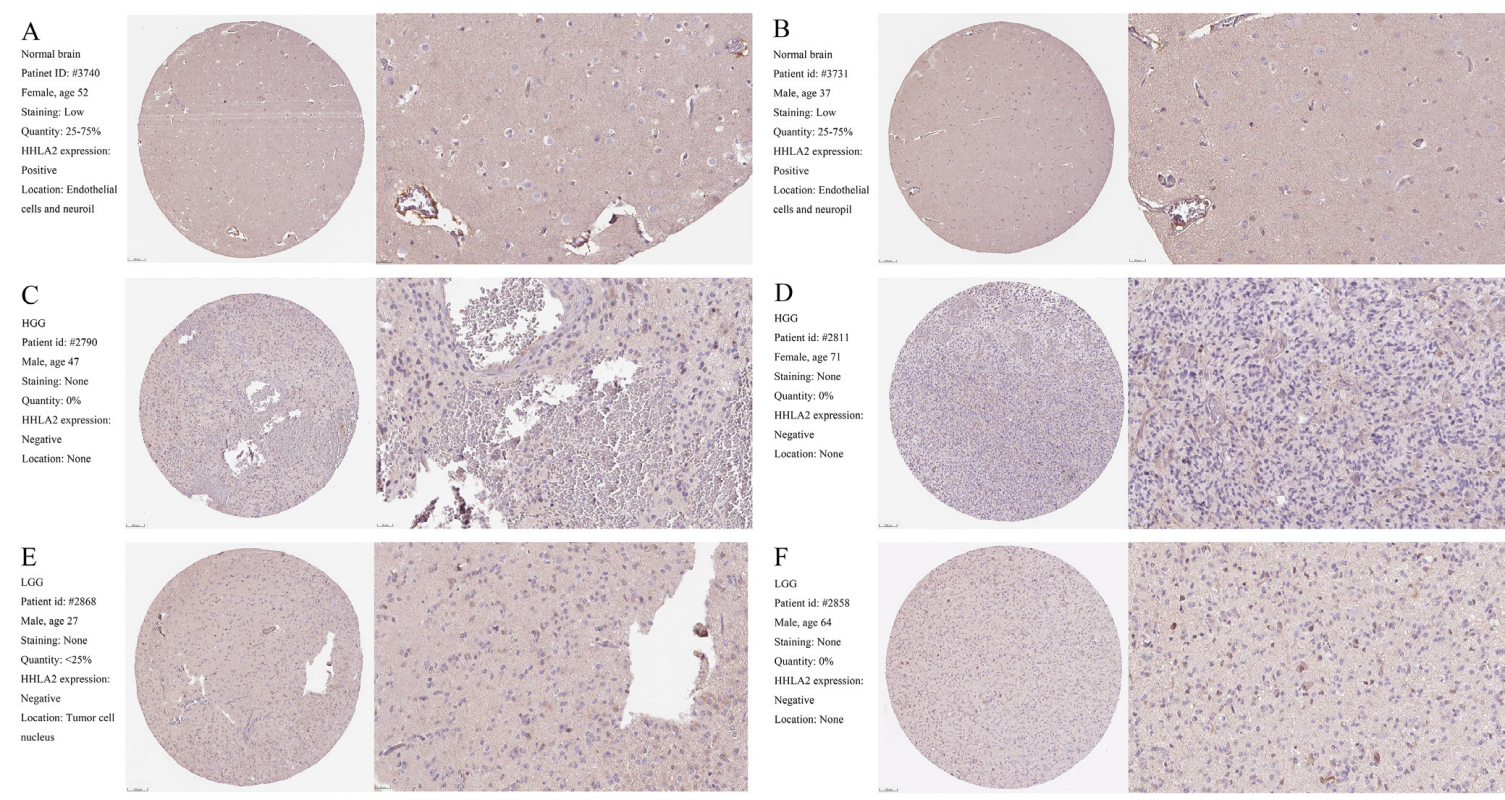
Representative specimens exhibiting HHLA2 IHC labeling pattern in normal brain, low-grade glioma, and high-grade glioma. HHLA2 IHC are presented. (A and B) Normal brain. (C and D) Low-grade glioma. (E and F) High-grade glioma. Scale bar, 100 and 25 µm, respectively.

